# PKG II reverses HGF-triggered cellular activities by phosphorylating serine 985 of c-Met in gastric cancer cells

**DOI:** 10.18632/oncotarget.9074

**Published:** 2016-04-28

**Authors:** Yan Wu, Xiaoyuan Yao, Miaolin Zhu, Hai Qian, Lu Jiang, Ting Lan, Min Wu, Ji Pang, Yongchang Chen

**Affiliations:** ^1^ Department of Physiology, School of Medicine, Jiangsu University, Zhenjiang City, Jiangsu Province, PR China; ^2^ Basic Medical Department, Changchun Medical College, Changchun City, Jilin Province, PR China

**Keywords:** type II cGMP-dependent protein kinase G, phosphorylation, c-Met, inhibition, gastric cancer cell

## Abstract

Previous studies showed that type II cGMP-dependent protein kinase G (PKG II) could inhibit the activation of epidermal growth factor receptor (EGFR). Both c-Met and EGFR belong to family of receptor tyrosine kinases (RTKs) and have high molecular analogy. However, the effect of PKG II on c-Met activation is unclear. This study was designed to investigate the inhibitory effect of PKG II on the activation of c-Met and consequent biological activities. The results from CCK8 assay, Transwell assay and TUNEL assay showed that HGF enhanced cell proliferation and migration, and decreased cell apoptosis. Activated PKG II reversed the above changes caused by HGF. Immunoprecipitation and Western blotting results showed that PKG II could bind with c-Met and phosphorylate its Ser985, and thereby inhibited HGF-induced activation of c-Met and MAPK/ERK and PI3K/Akt/mTOR mediated signal transduction. When Ser985 of c-Met was mutated to Alanine for preventing phosphorylation of this site, the blocking effect of PKG II on c-Met activation was annulled. When Ser985 of c-Met was mutated to Aspartic acid for mimicking phosphorylation of this site, HGF-induced activation of c-Met was prevented. In conclusion, the results indicated that PKG II could block c-Met activation via phosphorylating Ser985 of this RTK.

## INTRODUCTION

Gastric cancer is a significant public health problem worldwide due to its high incidence and mortality. Despite a universal decline in the incidence over the last several decades, this cancer remains the fourth most common cancer and is the second leading cause of cancer-related death worldwide [1, 2]. There is an urgent unmet need to improve treatment and outcome of this lethal disease. Research data showed that the tumorigenesis and development of gastric cancer was closely related to receptor tyrosine kinases (RTKs), including EGFR and c-Met [3, 4]. In gastric cancer tissues and cells, over-expression and high activity of some RTKs are closely related to the prognosis of the disease [5, 6]. Among the RTKs, EGFR is a key member and has been intensive studied. It has been confirmed that EGFR was abnormally high-expressed in gastric cancer cells and the inhibitors of this RTK, both small molecule chemical inhibitor and antibody against EGFR, have been used in treatment of gastric cancer [7, 8], suggesting that inhibiting activation of RTKs is a efficient way to treat cancers.

c-Met, also known as hepatocyte growth factor receptor (HGFR), is another key member of RTK super family and is expressed in epithelial cells of many organs, including the liver, the pancreas and the prostate [9, 10]. c-Met consists of three parts, including extracellular domain, trans-membrane domain, and intracellular domain. The extracellular domain provides binding site for HGF, the only ligand of c-Met [11]. The intracellular tyrosine kinase catalytic domain induces receptor activation via causing auto-phosphorylation of tyrosine on the receptor, including Tyr1234 and Tyr1235 which are docking sites for downstream signal transduction molecules [12]. There are also some serine/threonine residues on the intracellular domain and their phosphorylation plays role in regulating tyrosine kinase activity of the receptor. For example, phosphorylation of Serine 985 of c-Met could inhibit the tyrosine phosphorylation/activation of the receptor [13, 14]. The tyrosine phosphorylation/activation of c-Met can initiate downstream signaling of several transduction pathways, such as MAPK and PI3K/Akt mediated pathways, regulating cell proliferation, survival, motility and differentiation [15]. It was reported that dysregulation of c-Met was implicated in the development and progression of certain tumors, including gastric cancer [6, 16, 17]. The high expression and activity of c-Met in gastric cancer tissues and cells have been confirmed, indicating that inhibition of c-Met will also have significant role in treating gastric cancer [4].

Type II cGMP dependent protein kinase G (PKG II) is a serine/threonine kinase and has inhibitory effect on cell proliferation and metastasis but promote apoptosis, especially in tumor cells [18, 19]. Recent study from our laboratory showed that PKG II had an inhibitory effect on EGFR activation and consequently inhibited the EGF/EGFR induced MAPK, PLCγ and PI3K/Akt mediated signal pathways in a variety of tumor cells [20–23]. Additionally, PKG II could also inhibit the activation of some other RTKs, such as vascular endothelial growth factor receptor (VEGFR), platelet derived growth factor receptor (PDGFR) and insulin like growth factor-1 receptor (IGF-1R) [24]. Since RTKs exhibit a high level of conservation in their molecular structure [25, 26], we hypothesized that PKG II may also exert a similar inhibitory effect on c-Met. So, the inhibition and mechanism of PKG II on c-Met activation were investigated in this study.

## RESULTS

### PKG II inhibits HGF-induced changes of proliferation, migration and apoptosis activities in gastric cancer cell lines AGS and HGC-27

### c-Met expression is higher in AGS and HGC-27 cells

**Figure 1:**
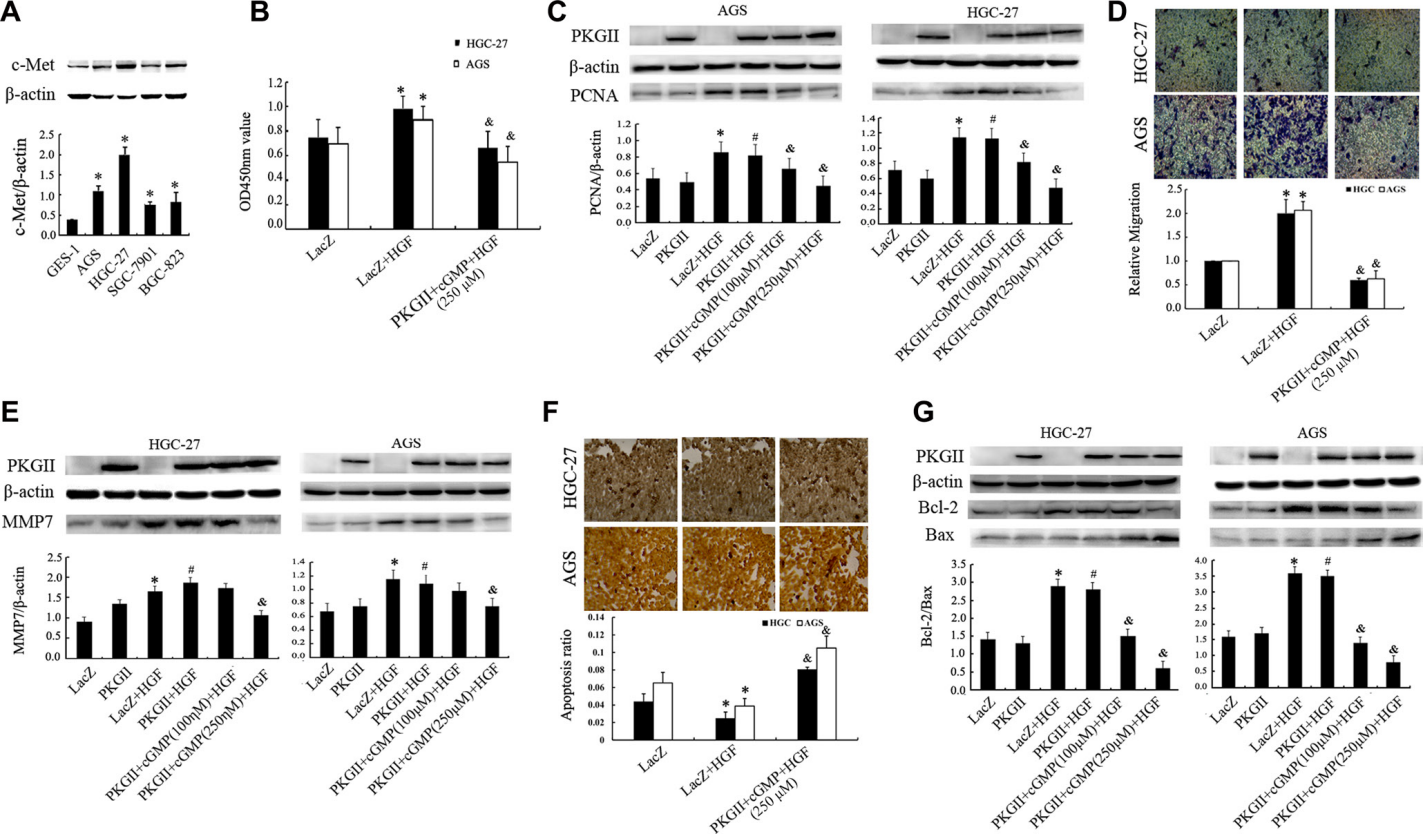
Analysis of the effect of PKG II on HGF-induced changes of proliferation, migration and apoptosis activities (**A**) The expression of c-Met in human gastric epithelial cell line GES-1 and gastric cancer cell lines AGS, HGC-27, SGC-7901 and BGC-823. (*P < 0.05, compared to GES-1 cell). (**B–G**) The detection of proliferation, migration, apoptosis activities and related protein expression. The AGS and HGC-27 cells were infected with either Ad-LacZ or Ad-PKG II, serum-starved for 12 h, stimulated with 8-pCPT-cGMP (100 or 250 μΜ) for 1 h, and then treated with HGF (50 ng/ml) for 24–48 h. (B) The relative proliferation activities were presented. (**C**) The expression of PCNA was detected by Western blotting with an anti-PCNA antibody. (D) The transwell migration assay was applied to detect the migration activity. The representative data showed the relative activities of migration. (E) The expression of MMP7 was detected by Western blotting with an anti-MMP7 antibody. (F) TUNEL method was applied to analyze the apoptosis activity, and the average ratio of apoptotic cells per field (magnification, × 200) was shown. (G) Detection of Bax and Bcl-2 protein by Western blotting in different groups. (*P < 0.05, compared to LacZ group; ^#^*P* < 0.05, compared to PKG II group; ^&^*P* < 0.05, compared to LacZ+HGF group, or PKG II+HGF group).

The expression c-Met is high in majority of tumor cells and the high expression makes it a potential marker for assessment of the degree of malignancy of gastric cancer [27]. To confirm that high expression of c-Met was also a bio-marker in cancer cell lines, the expression of c-Met was detected in normal gastric mucosa epithelial cell line and a variety of gastric cancer cell lines by Western Blotting. The results showed that compared with normal gastric mucosa epithelial cell GES-1, c-Met expression was higher in the gastric cancer cells, especially in AGS and HGC-27 cells (Figure 1A).

### PKG II inhibits HGF/c-Met induced proliferation of AGS and HGC-27 cells

Cell proliferation is an important characteristic of life activity, and excessive proliferation promote tumorigenesis. To investigate the role of HGF/c-Met in regulating proliferation of gastric cancer cells and the effect of PKG II on it, cell proliferation activity was analyzed by CCK8 kit, and the expression of proliferating cell nuclear antigen (PCNA) was detected by Western blotting in AGS and HGC-27 cells. The results from CCK8 kit analysis indicated that the proliferation was increased by stimulation with HGF (50 ng/ml, 12 h), while the increase of PKG II activity through infecting the cells with Ad-PKGII and stimulating the cells with cGMP efficiently prevented the HGF-induced proliferation (Figure 1B). Similarly, the HGF induced increase of PCNA expression was also reduced by activated PKG II (Figure 1C).

### PKG II inhibits HGF-triggered migration of AGS and HGC-27 cells

Cell migration is a central process in the development and maintenance of multicellular organisms, but errors during this process have serious consequences, such as tumor formation and metastasis. Vigorous migration is the deviant tendencies of tumor cells. The results from transwell migration assay showed that HGF treatment increased the migration activity of AGS and HGC-27 cells, and the increased expression and activity of PKG II efficiently inhibited the HGF-induced migration. (Figure 1D).

Matrix metalloproteinase-7 (MMP-7), as a member of the matrix metalloproteinase (MMP) family, is known to be involved in tumor metastasis and inflammatory processes. Hence, the expression of MMP7 can also reflect the invasion activity [28]. The results from Western blotting showed that HGF treatment induced a notable increase of MMP7 expression, and the increase of PKG II activity effectively decreased the HGF-induced expression of MMP7 (Figure 1E).

### PKG II reverses anti-apoptotic effect of HGF in AGS and HGC-27 cells

Apoptosis is the normal death that may occur in multicellular organisms. Apoptosis reduction promote the tumorigenesis and tumor progress [29]. TUNEL method was used to analyze the effect of HGF and PKG II on apoptosis of AGS and HGC-27 cells. The results displayed that HGF treatment reduced the apoptosis of the cells, and Ad-PKG II infection and 8-pCPT-cGMP treatment caused a significant increase of apoptosis of the cells, reversing the anti-apoptotic effect of HGF (Figure 1F).

Bcl-2 is an important anti-apoptotic protein and Bax is the protein promoting cell apoptosis [30]. The ratio of Bcl-2/Bax can well reflect the apoptosis activity. In this experiment, Western Blotting was used to detect the expression of Bcl-2 and Bax caused by treatment with HGF and PKG II. The results revealed that HGF treatment (50 ng/ml, 12 h) caused an increase in the expression of Bcl-2, but have no obvious effect on Bax expression. In other words, the ratio of Bcl-2/Bax was increased after HGF treatment. When the cells were infected with Ad-PKG II and treated with 8-pCPT-cGMP before being stimulated with HGF, the HGF-induced increase of Bcl-2 was abolished and an increase of Bax expression appeared, causing a decrease of the ratio of Bcl-2/Bax (Figure 1G). The above results suggested that PKG II promoted the expression of pro-apoptosis protein and reduced the expression of anti-apoptosis protein, thereby increasing the apoptosis of AGS and HGC-27 cells.

### PKG II blocks HGF-triggered signaling of MAPK/ERK mediated pathway

**Figure 2:**
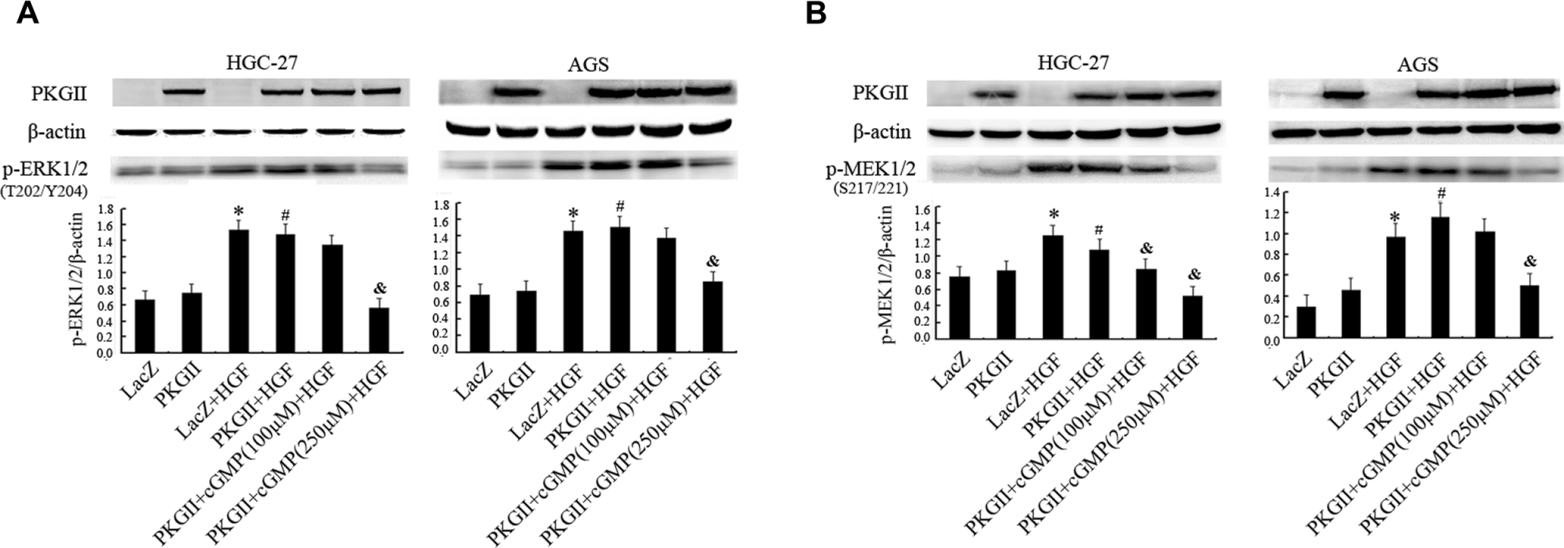
Analysis of the effect of PKG II on the activation of main components in MAPK/ERK pathway induced by HGF The AGS and HGC-27 cells were treated same as in Figure 1, excepting that the HGF (50 ng/ml) treatment is 10 min. (**A**) The phosphorylation of ERK1/2 in different group was detected by Western blotting with antibody against p-ERK1/2 (Thr 202/Tyr 204). (**B**) The phosphorylation of MEK1/2 in different group was detected by Western blotting with antibody against p-MEK1/2 (Ser217/221). (*P < 0.05, compared to LacZ group; ^#^*P* < 0.05, compared to PKG II group; ^&^*P* < 0.05, compared to LacZ+HGF group, or PKG II+HGF group).

The activation of c-Met can trigger the signal transduction of MAPK signal pathway, initiating cell proliferation and migration [31]. ERK1/2 are key members of MAPK family. Phosphorylation at both Thr 202 and Tyr 204 residues of its amino acid chain is required for full enzymatic activation of ERK1 [32]. Western blotting with antibody against p-ERK1/2 (Thr 202/Tyr 204) was used to detect the dual phosphorylation of ERK1/2. The results indicated that HGF (50 ng/ml) treatment for 10 min caused a dramatic increase of phosphorylation of ERK1/2. In the cells infected with Ad-PKG II and stimulated with 8-pCPT-cGMP and then treated with HGF, the phosphorylation level of ERK1/2 had no obvious increase (Figure 2A).

MEK1/2 is a member of the dual specificity protein kinase family, which acts as a MAPK/ERK kinase [33, 34]. Activity of MEK1/2 can be increased by phosphorylation of serine residues at 217 and 221 [34]. Western blotting with antibody against p-MEK1/2 (Ser 217/221) was applied to detect the activation of MEK1/2. The results showed that HGF (50 ng/ml) treatment caused obvious phosphorylation of MEK1/2, while the high expression and activity of PKG II blocked the phosphorylation (Figure 2B). These results revealed that PKG II could block HGF-induced signaling of MAPK/ERK mediated pathway.

### PKG II blocks HGF-induced signaling of PI3K/Akt/mTOR mediated pathway

**Figure 3:**
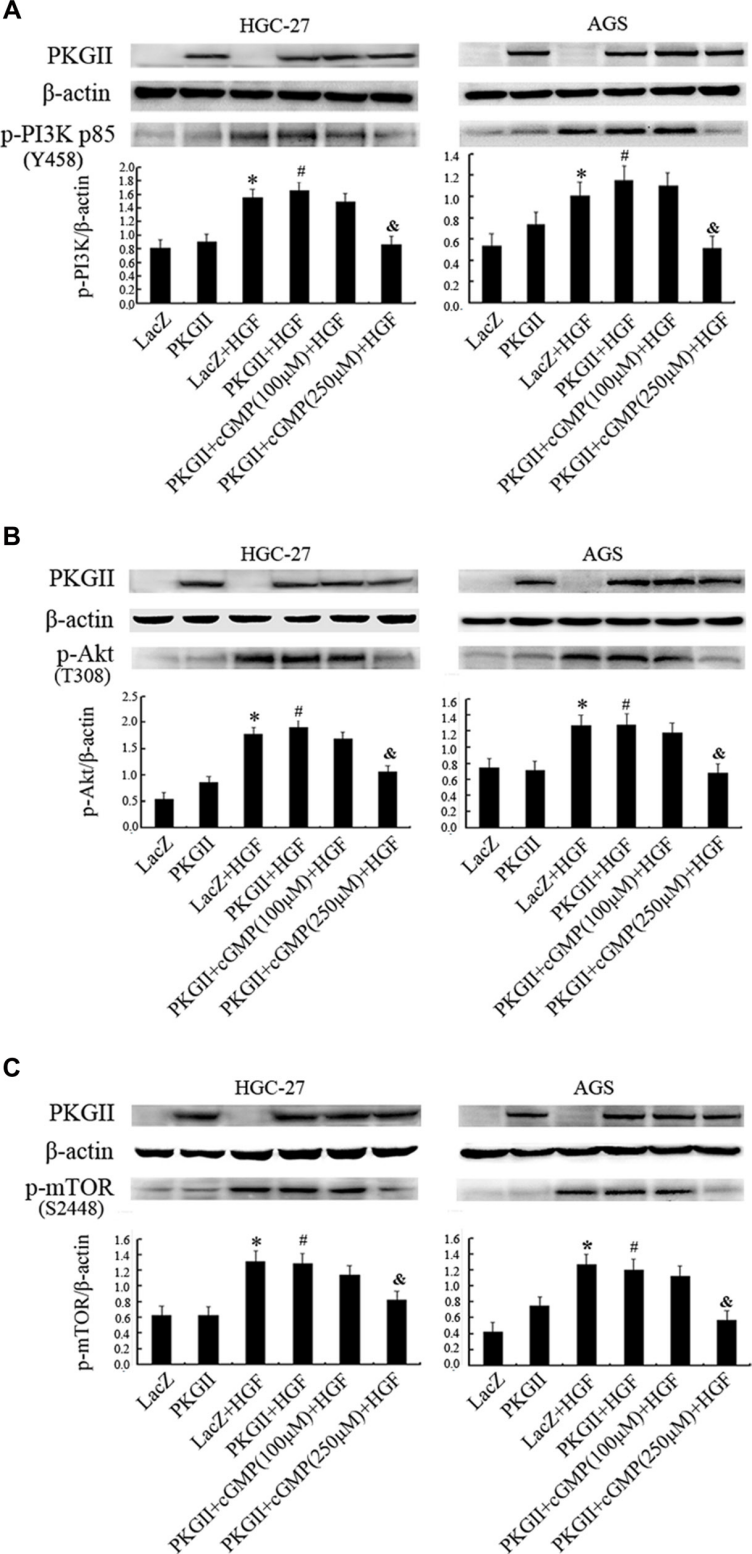
Analysis of the effect of PKG II on the activation of main components in PI3K/Akt/mTOR pathway induced by HGF The AGS and HGC-27 cells were treated with the same conditions as in Figure 2. (**A**) Western blotting was performed to detect the phosphorylation of PI3K p85 at Tyr 458 site in different groups. (**B**) The phosphorylation of Akt were detected by Western blotting with antibody against p- Akt (Thr 308). (**C**) Western blot analysis of Ser2448 phosphorylation of mTOR in different groups. (*P < 0.05, compared to LacZ group; ^#^*P* < 0.05, compared to PKG II group; ^&^*P* < 0.05, compared to LacZ+HGF group, or PKG II+HGF group).

The PI3K/Akt/mTOR mediated pathway is an important intracellular signaling pathway in regulating the cell cycle [35]. In multiple cancer tissues and cells, this pathway is overactive, reducing apoptosis and promoting proliferation. Activated PI3K can phosphorylate and activate Akt, localizing it in the plasma membrane [36]. Akt can subsequently activate downstream effects such as mTOR [36]. Therefore, we chose the corresponding antibodies to detect the phosphorylation/activation of PI3K, Akt and mTOR after HGF and PKG II treatments. The results revealed that HGF treatment (50 ng/ml, 10 min) caused a notable increase of PI3K p85α phosphorylation at Tyr458, Akt phosphorylation at Thr308, and mTOR phosphorylation at Ser2448. The increase of PKG II activity by infecting the cells with Ad-PKG II and stimulating with 8-pCPT-cGMP efficiently inhibited the HGF-induced phosphorylation/activation of the above proteins, suggesting that the activated PKG II could inhibit the transduction of PI3K/Akt/mTOR pathway induced by HGF (Figure 3A–3C).

### PKG II blocks HGF induced c-Met activation in AGS and HGC-27 cells

**Figure 4:**
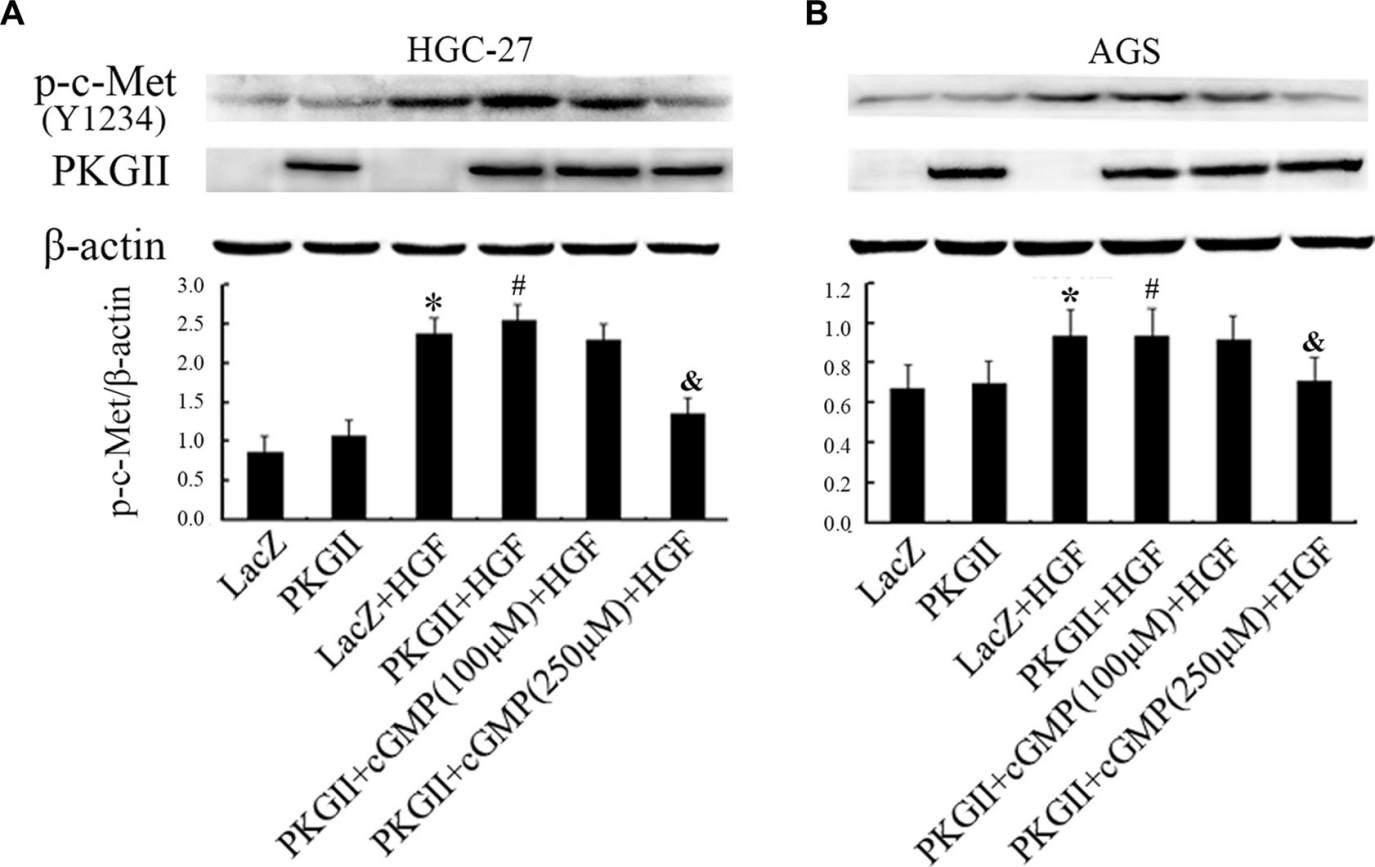
Analysis of the effect of PKG II on Tyr1234 phosphorylation of c-Met stimulated by HGF The AGS and HGC-27 cells were treated with the same conditions as in Figure 2. (**A**) The Tyr1234 phosphorylation level of c-Met in HGC-27 cells were detected by Western blotting and the representative data was shown. (**B**) Western blot analysis of Tyr1234 phosphorylation of c-Met in different groups of AGS cell.

Since the above results showed that PKG II inhibited HGF/c-Met-triggered multiple signal pathways and cell biological activities, suggesting that the initial inhibition site of PKG II might be at the upstream of the pathways, i.e. the receptor c-Met. We applied Western blotting with the antibody against p-c-Met (Tyr1234) to detect the c-Met activation under the HGF and/or PKG II treatments. The results revealed that the level of p-c-Met (Tyr1234) was increased distinctly in cells infected with Ad-LacZ and treated with HGF, and decreased in cells infected with Ad-PKG II, treated with cGMP and HGF, demonstrating that the increase of PKG II activity efficiently blocked the HGF-induced activation of c-Met. (Figure 4)

### PKG II prevents HGF-induced c-Met activation by phosphorylating c-Met at Ser985

### PKG II binds with c-Met and induces serine/threonine phosphorylation of c-Met

**Figure 5:**
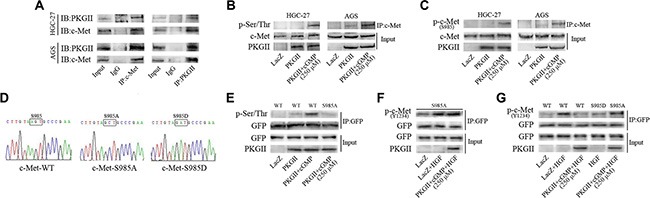
PKG II blocks HGF-induced c-Met activation by phosphorylating c-Met at Ser985 (**A**) Interaction between PKG II and c-Met. The AGS and HGC-27 cells were infected with Ad-PKG II, and then successively stimulated with 8-pCPT-cGMP (250 μΜ, 1 h) and HGF (50 ng/ml, 10 min). The cells were lysed and the lysates were immuno-precipitated with anti-c-Met antibody, anti-PKG II antibody and isotype-matched IgG, respectively. The precipitates were probed with anti-PKG II antibody (or anti-c-Met antibody), with 5% of cell lysate as protein input control. (**B**) PKG II increased pan-ser/thr phosphorylation of c-Met. The cells were infected with Ad-PKG II and then stimulated with 8-pCPT-cGMP (250 μΜ, 1 h). The cells were lysed and the lysates were immuno-precipitated with anti-c-Met antibody, and the precipitates were probed with anti-pan-Ser/Thr antibody. (**C**) PKG II induced Ser985 phosphorylation of c-Met. The cells were treated same as in panel B, and the precipitates were probed with anti-p-c-Met (Ser985) antibody. (**D**) The sequencing data of plasmid constructs c-Met-WT, c-Met-S985A and c-Met-S985D. (**E**) Ser985 was a PKG II-specific phosphorylation site in c-Met. The COS-7 cells were transfected with GFP-tagged plasmids of wild type c-Met or mutant c-Met-S985A, and then successively infected by Ad-PKG II for 24 h and treated with 8-pCPT-cGMP (250 μΜ) for 1 h. The cells were harvested and lysate was prepared. Immunoprecipitation with antibody against GFP was performed to precipitate wild type or mutant c-Met, and the pan-Ser/Thr phosphorylation in the precipitates were analyzed by Western blotting with anti-pan-Ser/Thr antibody. (**F**) HGF-induced Tyr1234 phosphorylation of mutant c-Met-S985A is not inhibited by PKG II. The COS-7 cells were treated as the above and then stimulated by HGF (50 ng/ml) for 10 min. The cell lysates were immuno-precipitated with anti-GFP antibody, and the precipitates were probed with anti-p-c-Met (Tyr 1234) antibody. (**G**) The COS-7 cells were treated same as in panel (F) The results showed that HGF-induced Tyr1234 phosphorylation was inhibited in c-Met-WT and c-Met-S985D, while not inhibited in mutant c-Met-S985A.

Co-immunoprecipitation method was applied to detect the binding between c-Met and PKG II. Both AGS and HGC-27 cells were infected with Ad-PKG II for 24 h and stimulated with 8-pCPT-cGMP for 1 h, and then the total cell lysate was immunoprecipitated with anti-c-Met antibody and probed with antibody against PKG II, and immunoprecipitated with anti-PKG II antibody and probed with anti-c-Met antibody, respectively. The Co-IP results showed that PKG II and c-Met bound together (Figure 5A). Next, the cells were treated as the above, and the cell lysate was immunoprecipitated with anti-c-Met antibody to isolate and concentrate c-Met. Western blotting with antibody against pan phospho-serine/threonine to detect the level of serine and threonine phosphorylation in the precipitated c-Met. The results displayed that serine/threonine phosphorylation level of c-Met was increased significantly in the cells treated with Ad-PKG II and 8-pCPT-cGMP, indicating that activation of PKG II caused serine/threonine phosphorylation of c-Met. (Figure 5B)

### Ser985 of c-Met is PKG II-specific phosphorylation site

It was found that the anti-p-c-Met (Ser985) antibody is the only available antibody to detect the serine phosphorylation of c-Met, and the prediction result from the Group-based Prediction System (GPS) software also showed that Ser985 is a potential site that PKG II act on c-Met. So we first analyzed the effect of PKG II on the Ser985 phosphorylation of c-Met. Cells were infected with Ad-PKG II and treated with 8-pCPT-cGMP, and then the total cell lysate was immunoprecipitated with anti-c-Met antibody and the phosphorylation level of c-Met at Ser985 was detected by Western blotting with antibody against p-c-Met (Ser985). It was found that there was an obvious increase of Ser985 phosphorylation of c-Met in the cells, suggesting that high expression and activity of PKG II increased Ser985 phosphorylation of c-Met (Figure 5C).

### PKG II fails to induce phosphorylation of Mutant c-Met S985A

On the basis of the above results, c-Met-S985A (Ser985→Ala) mutant, c-Met-S985D (Ser985→Asp) mutant and its wild-type (WT) counterpart in pIRES2-EGFP vector were created. In the c-Met-S985A mutant, 985 site could not be phosphorylated. In c-Met-S985D mutant, changing Serine 985 to Aspartic acid could simulate the phosphorylation of this site (Figure 5D). COS-7 cells were transfected with the recombinant vector and empty vector, and then infected with Ad-PKG II for 24 h and stimulated by 8-pCPT-cGMP for 1 h. At last the total cell lysate was harvested and immunoprecipitated with anti-GFP antibody, and then the serine/threonine phosphorylation was detected. The results showed that when the expression and activity of PKG II was increased, there was a marked increase of p-Ser/Thr level in cells transfected with c-Met-WT, but there was no increase of p-Ser/Thr level in the cells that were transfected with c-Met-S985A, indicating that Ser985 was the specific site that PKG II acted on c-Met. (Figure 5E)

### Mutant c-Met S985A annuls the inhibitory effect of PKG II on Tyr1234 phosphorylation of c-Met

To further confirm whether PKG II inhibit c-Met activation through Ser985, the effect of Ser985 mutation on Tyr1234 phosphorylation of c-Met was analyzed. COS-7 cells were sequentially transfected with mutant c-Met-S985A plasmid, infected with Ad-LacZ or Ad-PKG II, stimulated with 8-pCPT-cGMP, and treated with HGF for 10 min. The total cell lysate was harvested and immunoprecipitated with anti-GFP antibody, and then the Tyr1234 phosphorylation level was detected by Western blotting with anti-p-c-Met (Y1234) antibody. The results showed that PKG II had no obvious effect on HGF-induced Tyr1234 phosphorylation of c-Met, suggesting that the mutation of Ser985 to Ala could annul the inhibitory effect PKG II on Tyr1234 phosphorylation/activation of c-Met triggered by HGF (Figure 5F).

### Ser985 phosphorylation blocks Tyr1234 phosphorylation of c-Met

In order to further prove that the phosphorylation of Ser985 inhibited Tyr1234 phosphorylation of c-Met, the Ser985 was mutated to aspartic acid (c-Met-S985D) to simulate the phosphorylation of this site. The wild type c-Met-WT and mutant c-Met-S985A and c-Met-S985D plasmids were transfected into COS-7, and the cells were treated with Ad-PKG II, 8-pCPT-cGMP and HGF as described above. The total cell lysate was collected, ectogenic c-Met protein was precipitated with anti-GFP antibody, and then the Tyr1234 phosphorylation level was detected. The results showed that HGF treatment increased Tyr1234 phosphorylation of c-Met and PKG II inhibited this increase in cells transfected with c-Met-WT (WT). The above inhibition effect was not observed in cells transfected with c-Met-S985A (S985A), indicating that mutating Serine 985 to non-phosphorylated arginine interrupted the inhibitory action of PKG II on activation of c-Met. In addition, in cells un-infected with Ad-PKG II, mutant c-Met-S985D suppressed Tyr1234 phosphorylation of c-Met stimulated by HGF. These results suggested that Ser985 phosphorylation of c-Met could block the Tyr1234 phosphorylation, and PKG II promoted c-Met phosphorylation at Ser985, thereby blocked the tyrosine phosphorylation/activation of c-Met (Figure 5G).

## DISCUSSION

Our previous study demonstrated that PKG II could inhibit the activation of some key members of the RTK family, including EGFR, VEGFR, PDGFR and IGF-1R, and the consequent signal transduction in gastric cancer cells [24]. As another important member of RTK family, c-Met has similar molecular structure and is overexpressed in gastric cancer cells [37, 38]. Therefore, we hypothesized that PKG II may exert a similar inhibitory on the activation of c-Met. If this hypothesis is true, it will provide more evidence for confirming the broad inhibitory effect of PKG II on RTKs.

In the experiments, we investigated the effect of PKG II on HGF induced biological activities of gastric cancer cell, including the activation of c-Met, its downstream signaling of MAPK, PI3K/Akt and STAT mediated pathways, and the changes of proliferation, migration and apoptosis. First, we found that the activated PKG II suppressed the HGF-induced increase of proliferation and migration, and reversed the anti-apoptosis effect of HGF, suggesting that PKG II inhibited most HGF/c-Met enhanced biological activities. Further study showed that PKG II inhibited the HGF-induced signaling of MAPK/ERK and PI3K/Akt/mTOR mediated pathways, indicating that PKG II blocked the HGF/c-Met stimulated downstream signal transductions. The above results not only confirmed the inhibitory effect of PKG II on HGF/c-Met induced signaling and biological activities but also suggested that PKG II might inhibit the activation of c-Met.

Next, the mechanism through which PKG II blocked the activation of c-Met was explored. To confirm the direct inhibition of PKG II on the activation of c-Met, it was imperative to make clear whether PKG II bound with c-Met and caused phosphorylation of it. The Co-IP result showed that PKG II bound with c-Met. When c-Met was enriched by IP and detected by Western blotting with antibody against pan Serine/Threonine phosphorylation, the results showed that activated PKG II could increase serine/threonine phosphorylation of c-Met. These results indicated that PKG II could directly cause phosphorylation of c-Met.

And then, further study was carried out to identify the PKG II specific phosphorylation site of c-Met. In 1994, Gandino *et al.* found that PKC induced Ser985 phosphorylation of c-Met could inhibit the kinase activity of this receptor and block HGF-triggered signaling [39]. A few years later, Nakayama *et al.* reported that phosphorylation of Ser985 in c-Met inhibited tyrosine phosphorylation of this receptor [40]. Thus, we speculated that PKG II may block the c-Met activation through similar mechanism of PKC. To confirm this, we detected the effect of PKG II on Ser985 phosphorylation of c-Met and the results proved that activated PKG II increased the phosphorylation level of this site. Meanwhile, we used prediction software GPS to predict the potential PKG II specific serine/threonine phosphorylation site of c-Met. The results also showed that Ser985 site was a potential site through which PKG II phosphorylated c-Met. Thereby it was preliminarily elucidated that PKG II inhibited the activation of c-Met via phosphorylating Ser985. In order to further confirm the role of Ser985 phosphorylation in blocking activation of c-Met, we constructed the plasmid encoding wild type c-Met, and then mutated Ser985 to Alanine which could not be phosphorylated. The results showed that in cells transfected with plasmid encoding Mutant c-Met, PKG II did not cause serine/threonine phosphorylation of c-Met and failed to block HGF-induced c-Met phosphorylation at Tyr1234, indicating that PKG II inhibited c-Met activation through phosphorylating Ser985 of c-Met. At the same time, we also mutated Ser985 of c-Met to Aspartic acid, which simulated phosphorylation of this site, the result displayed that HGF-induced Tyr1234 phosphorylation of c-Met was abolished. The above results further confirmed that PKG II block Tyr1234 phosphorylation/activation of c-Met via phosphorylating Ser985 of the receptor.

In Conclusion, this study proved the inhibitory effect of PKG II on activation of c-Met and the consequent signal transduction and biology activities, and elucidated the mechanism that PKG II suppressed c-Met activation. Combining our previous research results, we summarize that PKG II exerts a wide inhibitory effect on the activation of key RTK members, including EGFR, VEGFR, PDGFR, IGF-1R and c-Met. Therefore, PKG II may act as a potential wide range inhibitor for the above RTKs. This will throw new train of thought to planning anti-cancer strategy and discovering anti-cancer drugs.

## MATERIALS AND METHODS

### Cell lines, antibodies and chemicals

Human gastric cancer cell line AGS and HGC-27, and African green monkey kidney fibroblast-like cell line, COS-7 were provided by the Institute of Cell Biology (Shanghai, China). Adenoviral vectors encoding the cDNA β-galactosidase (Ad-LacZ) and PKG II (Ad-PKG II) were kind gifts from Dr Gerry Boss and Dr Renate Pilz, University of California, San Diego, CA, USA. c-Met (NP_000236.2) Wild-type counterpart in pIRES2-EGFP vector were generous gifts from Dr Yi-Ching Wang, National Cheng Kung University, Tainan, Taiwan. Dulbecco's modified Eagle's medium (DMEM) and fetal bovine serum (FBS) were obtained from Gibco (Grand Island, NY, USA). The antibody against PKG II was from Abgent Biotechnology (San Diego, CA). The antibody against c-Met, p-Met (S985), RhoA, GFP, and horseradish peroxidase (HRP)-conjugated antibody against β-actin were obtained from Santa Cruz (Dallas, TX, USA). Rabbit anti-p-MEK1/2 (S217/221) was from Cell Signaling Technology (Danvers, MA). Mouse anti-Rac1 was from BD Biosciences (San Jose, CA). Rabbit anti-phosphserine/threonine was from Abcam (Cambridge, MA). Rabbit anti-p-Met (Tyr1234), p-ERK1/2 (Thr202/Tyr204), p-Akt (Thr308), p-PI3K p85 (Tyr458), p-mTOR (Ser2448), PCNA, Bax, Bcl-2, MMP-7 were from Bioworld Technology (St. Louis Park, MN). The horseradish peroxidase (HRP)-conjugated secondary antibodies were from Jackson ImmunoResearch Laboratories (West Grove, PA). The cellular permeable cGMP analog 8-pCPT-cGMP was from Calbiochem (San Diego, CA). HGF was from Sigma (St. Louis, MO). The BioepitopeR protein A+G agarose IP was from Bioworld Technology (St. Louis Park, MN). The cell transfection reagent Lipofectamin^™^ 2000 and E.coli BL-21DE3 were from Invitrogen (Carlsbad, CA). QuikChange Lightning Site-Directed Mutagenesis Kit was from Agilent Technologies (Santa Clara, CA). SanPrep Column Plasmid Mini-Preps Kit was from Sangon Biotech (Shanghai, China). Cell Counting Kit-8 (CCK8) was from R&S Biotechnology (Shanghai, China). *In Situ* Cell Death Detection kit (TUNEL Technology) was from Roche Diagnostics (Mannheim, Germany). Transwell Permeable Support was from Corning Incorporated (Corning, NY). Electrochemiluminescence (ECL) reagents were from Millipore (Billerica, MA).

### Cell culture and treatment

Human gastric epithelial cell line GES-1, african green monkey kidney cell line COS-7 and gastric cancer cell lines AGS, HGC-27, SGC-7901 and BGC-823 were cultured in DMEM supplied with 10% FBS and maintained at 37°C in a humidified incubator with 95% air and 5% CO_2_. The medium was changed every two days and the cells were sub-cultured at confluence. On the day before infection, cells were freshly planted at 70–80% confluence, and the infection with Ad-LacZ and Ad-PKG II for 24 h was performed. Then the cells were serum-starved for 12 h, followed by treatment with 8-CPT-cGMP (250 μm/ml) for 1 h and then with HGF (50 ng/ml).

### CCK8 assay

Ten thousand cells (in 150 μl complete DMEM) were seeded in each well of a 96-well plate. After attachment, the AGS and HGC-27 cells were infected with Ad-LacZ or Ad-PKG II for 24 h. The cells were then washed and serum-starved overnight. Thereafter, the cells were incubated with 8-pCPT-cGMP (250 μΜ) for 1 h, and then treated with HGF (50 ng/ml) for 24 h. Approximately 10 μl of CCK8 dye was added to each well, and the plate was incubated for 30 min. The optical density (OD) at 450 nm was measured using microplate reader. Data were presented as a percentage of the value of control group, with *P* < 0.05 considered to be significant.

### Transwell migration assays

Migration activity of AGS and HGC-27 cells were detected by transwell system. After trypsinization, 5 × 10^4^ cells were seeded into the upper chamber containing culture medium without FBS. Cell migration to the bottom side of membrane was induced by medium containing 10% FBS in the lower chamber for 12 h at 37°C in a tissue culture incubator. The cells remaining in the upper chamber were carefully removed with cotton swabs. Migrated cells on the bottom side of the membrane were fixed in 4% paraformaldehyde solution for 30 min, stained in Giemsa solution for 10 min, and then rinsed in water. The stained cells were subjected to microscopic examination under a light microscope. Migrated cells were counted in 5 randomly selected fields per insert, and the values were averaged. All experiments were performed with three replicates under each migration condition.

### Detection of apoptosis

TUNEL was performed with *In Situ* Cell Death Detection kit, following the manufacturer's instructions. Briefly, cells grown on 24-well plates were fixed and endogenous peroxidase activity was quenched with 2% H_2_O_2_ for 5 min. The cells were incubated with TUNEL reaction mixture (containing terminal deoxynucleotidyl transferase buffer, 1 mM Mn^2+^, and fluorescein-labled dUTP) in a humidified atmosphere at 37°C for 1 h. The cells were washed with PBS and incubated with anti-fluorescein antibody conjugated with horseradish peroxidase for 30 min. After rinsing with PBS, the cells were stained with DAB solution, washed with PBS and then covered by cover slide. Under microscope, cells exhibiting brown nuclear staining were considered to be apoptotic. For each well, five × 200 fields were randomly selected and the positive cells in the fields were counted. The average of the positive cell numbers was taken as value of one experiment. The assay was repeated 3 times.

### Plasmid constructs, cell transfection and infection

Point mutations Ser985→Ala (S985A), Ser985→Asp (S985D) were introduced using the QuikChange site-directed mutagenesis kit according to the manufacturer's protocol. The sequences of all generated constructs (WT, S985A, S985D) were confirmed by sequencing. For transfection, COS-7 cells were sub-cultured the day before the process and the transfection of the cells with plasmids was performed by using Lipofectamine 2000 (Invitrogen) according to the manufacturer's protocol. After the transfection for 6 h, infection with Ad-LacZ or Ad-PKG II was performed.

### Western blotting

Protein samples were subjected to SDS-PAGE (8–12%) gel according to the molecular size of the protein and transferred onto a PVDF membrane. The PVDF membranes were blocked using 5% (w/v) non-fat milk in TBS-T for 1 h at room temperature. The incubation with the primary antibody was at 4°C overnight, followed by incubation with the corresponding secondary antibodies at room temperature for 1 h, with three washes after each incubation. The signal was visualized using ECL detection reagents.

### Immunoprecipitation

The cells growing on 100-mm culture plate were washed two times with cold PBS and lysed by RIPA buffer (50 mM Tris-HCl pH 7.4, 1% Triton X-100, 1 mM EDTA,1 mM leupeptin, 1 mM phenylmethylsulfonyl fluoride, 10 mM NaF, 1 mM Na_3_ VO_4_) 48 h after transfection and/or infection. The supernatant was obtained by centrifugation (12,000 rpm, 10 min). The supernatant was mixed with the antibodies or matched immunoglobulin as a negative control for 8 h rocking at 4°C. Fresh protein G conjugated to agarose was then added, followed by 2 to 3 h rocking at 4°C. The reaction mixtures were centrifuged at 2,000 rpm for 1 min at 4°C. The supernatant was then discarded, and the pellet was washed four times with binding buffer and then resuspended with the same volume of 2 × SDS sample buffer. The precipitates were probed with antibodies against the protein.

### Statistical analysis

Data are expressed as the mean ± standard deviation. Statistical analysis was performed using a two-tailed ANOVA with SPSS statistical software (SPSS, Inc., Chicago, USA). *P* < 0.05 was considered to indicate a statistically significant difference.
